# Immunotherapy in Non-Small-Cell Lung Cancer Patients with Driver Alterations: A New Strategy?

**DOI:** 10.3390/cells11203280

**Published:** 2022-10-18

**Authors:** Natalia Krzyżanowska, Paweł Krawczyk, Kamila Wojas-Krawczyk, Tomasz Kucharczyk, Janusz Milanowski

**Affiliations:** Department of Pneumonology, Oncology and Allergology, Medical University of Lublin, 20-090 Lublin, Poland

**Keywords:** non-small-cell lung cancer, oncogenic driver alteration, immunotherapy, molecularly targeted therapy, immune checkpoint inhibitor

## Abstract

For many years, researchers have been trying to develop the most effective ways to fight lung cancer, which is the cause of the largest number of cancer-related deaths among men and women worldwide. The most advanced treatments for nearly all non-small-cell lung cancer (NSCLC) types include immunotherapy with immune checkpoint inhibitors (ICIs), mainly anti-programmed death 1/anti-programmed death ligand 1 monoclonal antibodies (anti-PD-1/PD-L1 mAbs) in monotherapy or in combination with other strategies. Despite significant advances, long survival is not achievable in most cases, so new solutions are constantly being sought. One of the questions raised by oncologists is the efficacy of ICIs in patients with molecular driver alterations, especially when the possibilities of using molecularly targeted therapies are exhausted (e.g., due to resistance to tyrosine kinase inhibitors). There are studies investigating this problem, but it is still poorly described. Among probable immunotherapy’ failures reasons, low immunogenicity of tumors with one driver mutation is listed. Nevertheless, in some cases, the therapy is efficient, and more research is required to establish the management of NSCLC patients with oncogenic driver abnormalities. The aim of this article is to review current discoveries in this matter.

## 1. Introduction

Despite the efforts of scientists and clinicians, lung cancer remains the leading cause of cancer-related deaths worldwide. The most common histological type of lung cancer is non-small-cell lung cancer (NSCLC) [1]. Diagnosis of the disease is often late and treatment options are scarce. Targeted therapy is one of the treatment strategies for advanced stages NSCLC patients with driver mutations. Oncogenic driver mutations are defined as genetic alterations responsible for both the initiation and maintenance of cancer. It often refers to genes encoding proteins essential for maintaining cellular proliferation, growth, and survival. Therefore, alterations of those genes may result in unrestricted proliferation and thereby lead to carcinogenesis and tumor progression. With the use of various methods, from immunohistochemistry (IHC) to next generation sequencing (NGS), patients are screened for such molecular changes. The most frequently occurring oncogenic driver alterations in NSCLC patients involve the following genes: *KRAS* (Kirsten rat sarcoma virus), *EGFR* (epidermal growth factor receptor), *ALK* (anaplastic lymphoma kinase), *ROS1* (ROS proto-oncogene 1), *BRAF* (proto-oncogene B-Raf), *MET* (mesenchymal-epithelial transition factor), *RET* (RET proto-oncogene), *NTRK 1-3* (neurotrophic tyrosine kinase type 1-3) and *HER2* (human epidermal growth factor receptor 2) [2]. Patients harboring alterations in those genes may benefit from personalized treatment based on the blockade of the abnormal proteins and signaling pathways. Registered therapeutics are summarized in Table 1 (Table 1). Presently, there is no registered targeted therapy for *HER2*-mutant NSCLC patients. Treatment regimens continue to evolve, but 5-year survival still occurs in only 20% of patients regardless of the disease stage, and in 10% of patients with metastatic disease [3].

On the other hand, in non-molecularly predisposed NSCLC patients, immunotherapy is introduced. The anti-tumor response is restored with the use of immune checkpoint inhibitors (ICIs)–monoclonal antibodies (mAbs) against negative co-stimulatory molecules. The most common targets within negative immune checkpoints are PD-1 (programmed death 1) on T cells and its ligand–PD-L1 (programmed death ligand 1) on tumor cells. Anti PD-1 agents used in NSCLC are pembrolizumab, nivolumab, and cemiplimab, and the anti-PD-L1 mAbs – atezolizumab and durvalumab. According to the registration rules, immunotherapy could be administered either in monotherapy or in combination with chemotherapy, based on the percentage of tumor cells (tumor proportion score, TPS) and/or immune cells with PD-L1 expression (combined proportion score, CPS), which is determined by IHC during qualification for the treatment. TPS ≥ 50% enables the introduction of ICIs in monotherapy; otherwise, patients may receive chemoimmunotherapy. A registered method of immunotherapy for NSCLC patients is also the combination of two ICIs: anti-PD1 (nivolumab) and anti-CTLA-4 (cytotoxic T lymphocytes antigen 4; ipilimumab) with chemotherapy. CTLA-4 is the immune checkpoint on lymphocytes.

All methods of immunotherapy could be used in advanced NSCLC patients after excluding the presence of at least basic genetic abnormalities in the *EGFR*, *ALK*, and *ROS1* genes since the low effectiveness of immunotherapy in such patients has been proven. This is due to the results of phase 3. clinical trials with nivolumab (CheckMate 057 study) and atezolizumab (OAK study). The effectiveness of ICIs was there compared to docetaxel in second-line treatment in NSCLC patients. Approximately 80 patients with mutations in the *EGFR* gene were enrolled in each study. The risk of death in patients with *EGFR* mutations was higher with immunotherapy compared to docetaxel (HR = 1.24; 95% CI: 0.71–2.18 in OAK study and HR = 1.18; 95% CI: 0.69–2.0 in CheckMate 057 study). In contrast, in patients without *EGFR* mutations, there was an evident benefit from immunotherapy compared to docetaxel. However, in phase 3 KEYNOTE 010 study, pembrolizumab showed an unclear benefit over docetaxel in reducing the risk of death in patients with *EGFR* mutations (HR = 0.88; 95% CI: 0.45–1.70) [14,15,16,17].

This article aims to review the rationale for introducing immunotherapy in oncogene-driven NSCLC patients on the basis of the literature data found. Still little is known about the benefit of including such therapy in groups of patients previously treated with targeted therapy and treatment-naive patients with driver genetic alterations, as well as therapy combining immunotherapeutics and molecularly targeted drugs.

## 2. Molecularly Targeted Therapies and Immunotherapeutics in NSCLC Patients—An Unobvious Combination

### 2.1. Immunotherapy Efficacy in Patients with Actionable Alterations in EGFR, ALK, and ROS1 Genes

The tyrosine kinase inhibitors (TKIs) have been pioneering molecularly targeted drugs. *EGFR* mutations and *ALK* or *ROS1* rearrangements are examined in the first instance as predictive factors, as they predispose patients to respond to TKIs (Table 1). Those medicines significantly improved the survival and life quality of NSCLC patients [2]. Unfortunately, the heterogeneity of tumors and constant exposure to new genetic alterations result in eventual development of either primary or required resistance to targeted therapy over time, including second- and third-generation TKIs. Among the reasons for this phenomenon, subsequent mutations/amplifications in genes encoding targeted proteins or upregulation of bypass signaling pathways have been pointed out [18].

Interest in immunotherapy for patients with mutations increased after reports of upregulation of PD-L1 expression in mutant *EGFR* and *ALK* cells, and improved survival in mutant *EGFR*-driven murine lung cancer models with PD-1 axis blockade [19,20,21]. However, presumptions about the efficacy of immunotherapeutics in *EGFR*/*ALK*-mutant patients have not been confirmed in the clinic. In the IMMUNOTARGET study, Mazieres et al. retrospectively studied the efficacy of ICIs monotherapy (anti-PD-1 or anti-PD-L1 agents used in second and subsequent lines of therapy) in patients with oncogenic alterations. Notably, no response in the *ALK*-rearranged group (*n* = 23) was observed. In *EGFR-*mutated group (*n* = 125), the overall response rate (ORR) was 12% and in *ROS1-*rearranged group (*n* = 7)—16%. There were some better results in groups of patients with other driver alterations, described in the sections below. Median progression-free survival (mPFS) was 2.1 months for patients with *EGFR* mutations and 2.5 months for *ALK* gene rearranged patients. The PFS correlated positively with PD-L1 expression. Patients with TPS ≥ 1% had 2.8 months of mPFS compared to patients without PD-L1 expression on cancer cells who reached 1.7 months of mPFS (*p* = 0.01). Moreover, differences were noted between groups with different types of *EGFR* mutations. The mPFS reached 1.4 months for patients with T790M mutation in exon 20 or complex mutations, 1.8 months for patients with deletions in exon 19, 2.5 months for patients with L858R substitution in exon 21, and 2.8 months for patients with other mutations (*p* < 0.001) (Table 2) [22].

Similar results have been obtained by Gainor et al. in a retrospective study on ICIs activity. Among 28 patients with *EGFR* mutations or *ALK* rearrangements, the response was observed in only 1 patient (3.6%), while in *EGFR* wild-type (WT) and *ALK*-negative/unknown patients, the response was reached in 7 of 30 patients (23.3%; *p* = 0.053). Additionally, PD-L1 expression and the presence of CD8^+^ tumor-infiltrating lymphocytes (TILs) were evaluated as independent predictive factors. Concomitant PD-L1 expression (TPS ≥ 5%) and high levels of CD8^+^ TILs (grade ≥ 2) were observed in only 1 pretreatment (2.1%) and 5 TKIs resistant (11.6%) *EGFR*-mutant patients and was not observed in any *ALK*-positive patients (Table 2) (Table 2) [23]. This could be the reason for the described outcome, as the absence of cytotoxic TILs in the tumor microenvironment (TME) may result in failure of immunotherapy, even with high PD-L1 expression on tumor cells [23]. Moreover, in the tumor microenvironment, cancer cell antigens must be successfully recognized so that the lymphocytes unblocked by ICIs are able to recognize and kill the cancer cell.

Immunotherapy (pembrolizumab) efficacy in TKIs-naive, *EGFR*-mutant patients (*n* = 10) has been evaluated in a phase 2 study (NCT02879994). No significant benefit was observed, even in patients with TPS ≥ 50%, and one death attributed to pneumonitis was reported (Table 2) [24]. In TKIs-naive patients, a combinatorial approach has been tested in phase 1/2 KEYNOTE-021 study, where NSCLC patients were treated with pembrolizumab and one of the EGFR inhibitors: erlotinib (cohort E, *n* = 12) or gefitinib (cohort F, *n* = 7). In cohort E, the safety profile was manageable, but in cohort F, grade 3/4 liver toxicity in 5 of 7 patients (71.4%) was observed and has led to discontinuation of the treatment in 4 patients. The therapy did not improve the ORR (which was 41.7%) when compared to trials results concerning monotherapy (Table 2) [25].

In the ATLANTIC study, researching durvalumab efficacy in patients with NSCLC and with different *EGFR/ALK* status, 12,2% of *EGFR*-mutated or *ALK*-rearranged patients (*n* = 111) with TPS ≥ 25% achieved the response. Among *EGFR*-wild*/ALK*-wild patients, the ORR was 16.4% in those who had TPS ≥ 25% (*n* = 265) and 30.9% in those who had TPS ≥ 90% (*n* = 68). Although the study results did not lead to the registration of durvalumab, the authors highlighted the fact that in *EGFR*-mutated patients with ≥25% of tumor cells expressing PD-L1 the activity of durvalumab was visible and further investigation is necessary (Table 2) [26].

Retrospective Lau et al. study on ICIs effectiveness in NSCLC patients with *EGFR* and *HER2* mutations has also revealed differences between distinct *EGFR* alterations. The ORR for *EGFR*-sensitizing mutations (*n* = 28) was 11% and for *EGFR* exon 20 mutations (*n* = 6) it was 50%. The mPFS was also favorable for patients with exon 20 mutations (4.8 months versus 1.7 months in patients with other mutations) (Table 2) [27].

Choudhury et al. evaluated response to immunotherapy in patients with *ROS1*-rearranged NSCLC either in monotherapy (*n* = 28) or in combination with chemotherapy (*n* = 11). The time to treatment discontinuation (TTD) lasted 2.1 months and the ORR was 13%, which is consistent with the IMMUNOTARGET study results. However, in the chemoimmunotherapy group the TTD lasted 10 months and the ORR was 83%, which is a distinctive result. Additionally, they observed low or no PD-L1 expression and low TMB in most patients (Table 2) [28]. Such outstanding outcome is encouraging, and it would be reasonable to compare chemotherapy and chemoimmunotherapy in patients with targetable mutations.

Immunotherapy for advanced-stages NSCLC patients is not the only use of ICIs. A recent study showed shortened disease-free survival after administration of the combination of neoadjuvant immunotherapy and chemotherapy in patients with *EGFR* exon 20 insertion or *MET* exon 14 skipping mutation, despite achieving the major pathological response in 3 of 4 patients with targetable driver mutations [38]. In contrast, clinical trials on neoadjuvant EGFR-TKIs provide favorable data in early stage *EGFR*-mutant NSCLC [39]. As for these alterations, more studies are required to determine which approach is the most effective, but targeted therapy appears to be the most favorable so far.

Generally, *EGFR* mutations, *ALK*, and *ROS1* rearrangements do not appear to predispose patients to immunotherapy. However, it must be taken into consideration that the data on the use of ICIs in patients with *ALK* and *ROS1* rearrangements is relatively scarce. It has been speculated that the lack of efficacy may be related to the low immunogenicity of *EGFR*- and *ALK*-mutant tumors. Despite the aforementioned assumptions about the upregulation of PD-L1 expression in *EGFR*-mutant tumors, PD-L1 status in this group is still questioned. Recent studies are leaning towards lower PD-L1 expression in *EGFR*-mutant tumors in comparison to smoking-related cancers, which are well infiltrated with T cells, have higher tumor mutational burden (TMB), and longer survival [40,41,42]. Even with high PD-L1 expression on cancer cells, the absence of cytotoxic TILs in TME may result in failure of immunotherapy. Regarding TMB, it has been stated that a high number of mutations results in neoantigen exposure, and thus, high TMB enhances tumor immunogenicity. Indeed, high TMB has been identified as a potential predictor of ICIs therapy [43,44]. The combinatorial approach using both immunotherapy and TKIs does not seem to be feasible as well, due to serious adverse events, visible also in sequential therapy [45,46]. Nonetheless, certain patients benefit from ICIs therapy; therefore, further investigation is required to discover the mechanisms underlying the response to the treatment.

### 2.2. Dualistic Effect of KRAS and BRAF Mutations on the Immune System and the Efficacy of ICIs

*KRAS* mutations are, along with *EGFR* alterations, the most frequent type of alterations in NSCLC patients (approximately 30%). They are almost only detected in lung adenocarcinoma (LUAC) and are associated with smoking [47]. Unlike in other mutant lung cancers, in *KRAS*-mutant tumors, additional genomic alterations frequently occur. The most common co-mutations involve *TP53* (tumor protein p53), *STK11* (serine/threonine kinase 11), and *KEAP1* (kelch like ECH associated protein 1) [48]. Although attempts are continually being made to find a relevant drug, there are no registered effective drugs targeting mutant KRAS, besides recently registered sotorasib for locally advanced or metastatic NSCLC patients with *KRAS* G12C mutation, who have received at least one prior systemic therapy, based on the CodeBreaK 100 study (Table 1) [49]. Additionally, preclinical and clinical data regarding another KRAS G12C inhibitor, adagrasib (MRTX 849), is promising. Adagrasib has earned a breakthrough therapy designation from the FDA based on findings from the phase 2 KRYSTAL-1 trial [50,51].

Considering the lack of targeted therapy and underdetermination of other treatment options, immunotherapy is being investigated. In the IMMUNOTARGET study mentioned above, the highest proportion of partial or complete responses was observed in the *KRAS*-mutant patients (*n* = 271). The ORR reached 26%, the mPFS lasted 3.2 months and was positively correlated with PD-L1 expression. Patients with PD-L1 TPS ≥ 1% reached 7.2 months of mPFS, whereas patients without PD-L1 expression obtained 3.9 months of mPFS. (*p* = 0.01). The outcomes were consistent with ICIs registration trials [22]. Interestingly, Skoulidis et al. reported that *TP53*-mutant adenocarcinomas were characterized by high levels of PD-L1, PD-1, and CTLA-4 expression and CD3^+^, CD8^+^, and CD45RO^+^ lymphocytes infiltration, whereas *STK11* co-mutation was associated with low levels of immune markers and mostly immune inertia, with low T-cell infiltration [48].

Kadara et al. had examined early stages LUACs and discovered that tumors with *STK11* mutations exhibited relatively low levels of infiltrating CD4^+^/CD8^+^ T-cells and, again, *TP53*-mutant cancers showed elevated PD-L1 TPS [52]. Moreover, in the analysis of LUAC samples from The Cancer Genome Atlas and GEO repository conducted by Dong et al., *KRAS* and *TP53* co-mutated subgroup demonstrated significantly higher PD-L1 expression than other co-mutation types and high intensity of CD8^+^ TILs infiltration. This was further confirmed by IHC staining of LUAC specimens. The authors also emphasized that the co-mutated subgroup showed higher mutational loads than other groups [53]. Moreover, it has been proven that patients with nonsquamous *STK11*-mutant NSCLC are less likely than *STK11* wild-type patients to respond to durvalumab monotherapy or durvalumab in combination with tremelimumab therapy (anti-CTLA-4 mAb), and their tumors show increased expression of genes and cytokines that activate STAT3 signaling pathway [54]. This allows to assume that the effectiveness of ICIs in patients with *KRAS* and *STK11* mutations may be low, and the researchers have confirmed these speculations in the clinic. Clinical outcome was inferior in patients with *STK11* mutation in multiple *KRAS*-cohorts of LUAC patients. The ORR in this group was 7.4%, whereas in the group with *TP53* mutation the ORR reached 35.7%. *TP53* co-mutation was favorable also in patients without PD-L1 expression (Table 2) [29].

The effectiveness of combined treatment with KRAS inhibitors and immunotherapy is being investigated in numerous clinical trials. In phase 1b study CodeBreaK 101, sotorasib is used in combination with other anticancer therapies (including anti-PD-1 mAbs) in patients with advanced solid tumors harboring *KRAS* G12C mutation [55]. Moreover, KRYSTAL-7 is a phase 2 study designed to further evaluate the clinical activity of adagrasib in combination with pembrolizumab administered as first-line treatment for patients with advanced NSCLC harboring a *KRAS* G12C mutation [56].

Mutations in *BRAF* gene occur in 3–4% of NSCLC patients and V600E mutation constitutes a great proportion of these alterations (approximately 50%) [57]. In such cases, BRAF inhibitor – dabrafenib in combination with MEK inhibitor–trametinib is introduced (Table 1) [9]. Considering the heterogeneity of non-V600-mutant NSCLC and scarce clinical data, management of patients harboring this type of mutation is challenging. According to Dudnik et al., *BRAF* mutation is connected with higher PD-L1 expression than in overall population of NSCLC patients, low or intermediate TMB, and microsatellite stability [30].

In the *BRAF*-mutated subgroup (*n* = 43) of the IMMUNOTARGET study, the ORR was 24% and the mPFS reached 3.1 months, similarly to the results in the *KRAS*-subgroup. Additionally, the lowest rate of progressive disease (PD) was observed in this group and the mPFS was significantly higher in smokers than in never smokers (4.1 vs. 1.9 months, *p* = 0.03). The cause may be sought in higher TMB in smokers. There was a difference in mPFS between patients harboring V600E mutation and patients with other *BRAF* mutations, but it was not statistically significant (1.8 months vs. 4.1 months, *p* = 0.20) (Table 2) [22]. Dudnik et al. reported that ICIs had favorable activity both in V600E-mutant (*n* = 12) and non-V600E-mutant patients (*n* = 10) as the ORR reached 25% and 33%, respectively (*p* = 1.0). The mPFS was 3.7 months in patients with V600E mutation and 4.1 months in patients with non-V600E mutations. Numerically, results in patients with mutations other than V600E seem to be slightly better but they were not significant (Table 2) [30].

Guisier et al. included 26 NSCLC patients with *BRAF* V600E mutation and 18 with non-V600E alteration in their study of ICIs efficacy in NSCLC and the ORR was 26% and 35%, respectively. The mPFS was 5.3 months in patients with V600E mutation and 4.9 months in patients with non-V600E mutation (Table 2) [31]. The outcomes in the studies mentioned above were mainly consistent with NSCLC unselected study groups and it appears that in V600E-mutant patients dabrafenib and trametinib combination is a more appropriate option hitherto. As for non-V600E mutation, the role of immunotherapy remains undetermined, but in the absence of other treatment options, it seems worth considering.

Spiegel et al. have performed comprehensive genomic profiling on lung cancer samples and reported that TMB was low in lung cancer harboring oncogenic driver mutations, but *BRAF-* (especially non-V600E) and *KRAS*-mutant patients were exceptional with a greater percentage of patients with high TMB. Median TMB values were as follows: 4.5 for *EGFR* mutation; 3.1 for *ALK/ROS1* fusion; 6.2 for *MET* ex. 14 alteration; 9.7 for *BRAF* mutation; and 10.3 for *KRAS* mutation (*p* < 0.001 in all groups in Wilcoxon signed-rank test vs. *KRAS*). Furthermore, high TMB was correlated with high DNA repair deficiency, another candidate for ICIs response predictor [58,59]. Singal et al. analysis of the clinogenomic database showed differences regarding TMB among distinct driver mutations. Alterations in *EGFR*, *ALK*, *ROS1*, and *RET* were associated with significantly lower TMB than WT cases (mTMB for *EGFR-*mutant: 3.5 vs. WT: 7.8; *ALK-*mutant: 2.1 vs. WT: 7.0; *ROS1-*mutant: 4.0 vs. WT: 7.0, and *RET*-mutant: 4.6 vs. WT: 7.0). Alterations in *PIK3CA* and *KRAS* were associated with significantly higher TMB (mTMB: *PIK3CA-*mutant: 8.7 vs. WT: 7.0; *KRAS-*mutant: 8.4 vs. WT: 6.1). All differences were statistically significant (*p* < 0.05) [60]. Considering the above, the TME and genetic features influencing TME are certainly worthy of further investigation to formulate the best treatment standards.

*KRAS*-mutant and *BRAF*-mutant patients constitute an interesting group regarding the response to ICIs. Undoubtedly, they may benefit from immunotherapy, but the implementation of ICIs in this group requires more research on molecular characteristics underlying the response. Nevertheless, patients harboring *KRAS* coupled with *TP53* mutation appear to be the most suitable target group for immunotherapy among all studied driver mutations.

### 2.3. Difficult Cases: Patients with MET, HER2, RET, and NTRK Abnormalities

As new mutations are being described, new TKIs are continually being developed. Durable response with MET, NTRK, and RET inhibitors is achievable, although as for all the mutations described in this paper, it is questionable whether immunotherapy can be used when the options for targeted therapies have been exhausted. Unfortunately, there is no registered targeted therapy for *HER2*-mutant NSCLC patients yet. An investigation on ICIs use in those patients is all the more important now.

Mazieres et al. analyzed ICIs efficacy also for patients with *MET* amplifications or exon 14 skipping mutations (*n* = 36). The mPFS was 3.4 months and the ORR was 16% in this group of patients. Half of the patients had PD, which was a relatively low proportion (Table 2) [22]. In the aforementioned Guisier et al. study, the ORR for the subgroup of patients with *MET* abnormalities (*n* = 30) reached 35.7% and the mPFS was 4.9 months, which is a better outcome than in other studies, but the authors outline that a great percentage of patients had high PD-L1 expression status and relatively low number of treatment lines received before immunotherapy (Table 2) [31]. Sabari et al. analyzed *MET* exon 14-altered lung cancer patients (*n* =  24) and response to ICIs. They stated that the ORR was 17% and the mPFS was 1.9 months. They did not find a correlation between response to ICIs and PD-L1 expression (TPS ≥ 50% and < 50%) or high TMB. The median TMB was lower than in unselected NSCLC population (Table 2) [32].

Patients with *HER2* mutations were also enrolled in the IMMUNOTARGET study (*n* = 29) and the outcomes were not entirely favorable: in this group of patients, the ORR was 7%, PD occurred in 67% of patients and the mPFS was 2.5 months. As it was in the *BRAF*-subgroup, the PFS correlated with smoking status (3.4 months for smokers vs. 2.0 months for never smokers, *p* = 0.04) (Table 2) [22]. Guisier et al. also provided analysis for *HER2*-mutant patients (*n* = 23). The ORR was 27.3% and mPFS lasted 2.2 months. (Table 2) [31]. In Lau et al. analysis, *HER2*-mutant patients (n = 14) achieved 27% ORR and mPFS was 3.6 months. The outcome was better than in patients with *EGFR* mutations other than exon 20 alterations (Table 2) [27]. Saalfeld et al. retrospectively evaluated outcomes of *HER2*-mutant NSCLC patients who were treated with immunotherapy or chemoimmunotherapy in the first-line setting (*n* = 27) or with ICIs in monotherapy as second or subsequent lines (*n* = 34). In treatment-naive patients receiving chemoimmunotherapy, the ORR, mPFS, and OS rate at 1 year were 52%, 6 months, and 88%, respectively. In the second or subsequent lines, ICIs monotherapy resulted in ORR of 16%, mPFS of 4 months, and median OS of 10 months (Table 2) [33]. Favorable results in the combination therapy group encourage further research on this approach.

In the IMMUNOTARGET study, only 6% of patients with *RET* rearrangements achieved partial or complete response to ICIs therapy, and 75% had PD. The mPFS was 2.1 months (although the study group was small, *n* = 16). These were the second most unfavorable outcomes in this study, right after the results in the subgroup of patients with *ROS1* rearrangements (Table 2) [22]. In Guisier et al. analysis, the *RET*-rearranged group (*n* = 9) achieved an ORR of 37.5% (3 out of 8 evaluable patients), and the mPFS in this group was 7.6 months (Table 2) [31]. Hegde et al. have conducted a study, in which the TTD due to progression has been determined in patients with *RET*-mutant malignancies, including NSCLC. Overall, the median TTD was longer in patients who received non-ICI therapy than in those who received ICIs. This was also true for the 29 patients with NSCLC, but in this case, it was not statistically significant (9.3 months vs. 3.4 months, *p* = 0.16). Even in patients with strong PD-L1 expression (*n* = 3), who received ICIs, TTD of less than 2 months was observed in two of them. Data on TMB and MSI was available in some patients and all of them had low TMB and were microsatellite-stable (Table 2) [34].

*NTRK1*, *NTRK2*, and *NTRK3* are genes encoding tyrosine kinase receptors and their fusions with other genes are frequent in some rare cancer types (e.g., secretory breast carcinoma), but in NSCLC the prevalence of these alterations is approximately 0.1–1% [61]. The treatment strategy for this type of cancer is tissue agnostic drugs used in patients with *NTRK* fusions, regardless of tumor type (Table 1) [10]. So far, only isolated case reports on the efficacy of immunotherapy in patients with *NTRK* fusions have been described. Two patients were included in Dudnik et al. study and one of them achieved a partial response. However, the mPFS and mOS were not reached in these two patients (Table 2) [35]. A patient with LUAC, harboring novel *NTRK* fusion (*NCOR2-NTRK1*), was treated with camrelizumab (anti-PD-1 mAb) and PD was observed, despite high TMB and PD-L1 positivity (20–30% of tumor cells). However, partial response for at least 5 months was achieved after switching the treatment option to larotrectinib (Table 2) [36]. Rosen et al. performed analysis on patients with TRK fusion, and one patient with LUAC, receiving ICI, achieved stable disease (Table 2) [37]. Undoubtedly, there are still many unanswered questions about immunotherapy in these patients.

### 2.4. Tertiary Lymphoid Structures: Prognostic and Predictive Potential

Tumor immune-microenvironment is deeply researched at the moment, and it comes across as the key to a greater understanding of immunotherapy response determination. As mentioned above, distinct mutations correlate with different levels of PD-L1 expression and CD8^+^ cell infiltration. Another intriguing TME feature is the presence of tertiary lymphoid structures (TLS). TLS are interesting components of tumor site, as they have a structure similar to lymph nodes (although they are not encapsulated) and they are induced in chronic inflammation, such as autoimmune diseases, persistent infections, organ transplantation rejection, and cancer. Tumor-infiltrating cells are dispersed, whereas in TLS, they are clustered in particular structures. Cell populations that constitute TLS, most frequently described in the literature, involve B cells, T cells, and mature dendritic cell-lysosomal associated membrane protein (DC-LAMP)^+^ dendritic cells (DCs). The prognostic value of TLS is broadly investigated in various cancers, and some studies have proven TLS to have a positive prognostic value, e.g., in melanoma, colorectal cancer, and lung cancer [62]. Rakaee et al. used artificial intelligence for TLS assessment in NSCLC histology images. No association between TLS and TMB was observed, but *EGFR*-mutated patients had a significantly lower number of TLS compared to the wild type. Patients with ≥0.01 TLS/mm^2^ had a significantly higher ORR (32% vs. 22%, *p* = 0.03), a longer mPFS (4.8 vs. 2.7 months, *p* = 0.004), and an improved median OS (16.5 vs. 12.5 months, *p* = 0.008) [63].

Other researchers have also correlated driver alterations with some TLS characteristics in NSCLC, on the assumption that DCs and other cells densities at the tumor site are treated as TLS indication. Biton et al. performed broad immune profiling in LUAC patients and concluded that the presence of *TP53* mutations without co-occurring *STK11* or *EGFR* alterations, independently of *KRAS* mutations, identified the group of tumors with the highest CD8^+^ T-cell density and PD-L1 expression. They determined DC-LAMP^+^ DCs density as the reflection of TLS presence and in *STK11*-mutated patients, this parameter was low [64]. DC-LAMP^+^ DCs are described as mature DCs, and it appears that they are responsible for enhancing anti-tumor immunity by tumor-antigen presentation; therefore, their presence at the tumor site is potentially favorable. The aforementioned results tend to confirm that *STK11*- and *EGFR*-mutant tumors are immunologically suppressed and *TP53* mutation characterizes well-infiltrated tumors which are predisposed to answer to ICIs. Although there is little data regarding TLS predictive potential in NSCLC, there are signs that determining TLS may be useful in predicting survival and response to immunotherapy in this type of cancer.

## 3. Summary

The search for NSCLC treatment optimization continues and new approaches are being tested. One of them is to introduce immunotherapy in patients with oncogenic driver mutations as resistance to targeted therapy occurs. Scientists are at the beginning of the journey to determine the full potential of immunotherapy, yet some conclusions can already be drawn at this stage.

Various targeting mutations appear to imply distinct tumor biology and susceptibility to therapies. The clinical response to ICIs varies depending on the driver mutation: in *KRAS*- (especially with *TP53* additional mutation) and *BRAF* non-V600E-mutant tumors, clinical benefit from the use of ICIs is achieved, while *EGFR*-, *ALK*-, *ROS1*-, *MET*-, *HER2*- and *RET*-mutant tumors usually do not respond for immunotherapy. Preliminary data indicate that the use of ICIs in the majority of driver-mutated oncogenic patients is not recommended and the treatment with TKIs or other inhibitors remains the therapy of choice in those NSCLC patients. In most of the reports, researchers conclude that immunotherapy may be considered solely after the exhaustion of targeted therapies and, in some cases, chemotherapy. However, the combination of ICIs and chemotherapy showed favorable outcomes in some studies which included patients with driver alterations. This approach certainly deserves consideration. Differences are also observed among groups with various types of mutations of the same gene and among groups with different co-occurring alterations. Those results highlight the necessity for broad molecular diagnostics in patients diagnosed with NSCLC. This could be achieved, for instance, by the use of the NGS methods. Presented results support the predictive value of PD-L1 expression and TMB, as a high value of these parameters is correlated with more beneficial outcomes.

The studies are still definable as preliminary, they are mainly retrospective, the groups are heterogeneous, and in large part not numerous, especially in the case of rare mutations. Moreover, patients receive immunotherapy in the first, second, or subsequent lines of treatment, thus it is critical to conduct a more thorough investigation. Undoubtedly, much research is still required on immunological and genetic changes that occur in TME during the course of the disease and various therapies. Large prospective studies and investigations of new predictive factors are essential. Immunotherapy is also still evolving and, as new therapeutic agents are being developed, presumably more clinical studies will be conducted. There are still many uncertainties, but the direction of future research has been defined and it appears that the path to the use of immunotherapy in patients with oncogenic dependencies, although limited, is not closed.

## Figures and Tables

**Table 1:** Oncogenic driver genes and corresponding targeted therapies for NSCLC patients currently used in the clinic (approved by FDA and/or EMA).

Altered Gene	Product of the Gene	Alterations Prevalence (Advanced LUAC)	Registered Therapeutics	Ref.
*EGFR* (ex. 18–21)	receptor tyrosine kinase	30.3%	erlotinib, gefitinib, afatinib, dacomitinib, osimertinib	[4,5]
*EGFR* (ex. 20)	receptor tyrosine kinase	0.7% (all stages NSCLC)	mobocertinib, amivantamab	[5,6]
*ALK*	receptor tyrosine kinase	4.4%	crizotinib, ceritinib, alectinib, brigatinib, lorlatinib	[5,7]
*ROS1*	receptor tyrosine kinase	1.9%	crizotinib, entrectinib	[5,8]
*BRAF*	serine/threonine kinase	5.5%	BRAF inhibitor dabrafenib + MEK inhibitor trametinib (for *BRAF* V600E mutation)	[5,9]
*NTRK1-3*	receptor neurotrophic tyrosine kinase	0.1–1%	entrectinib, larotrectinib	[5,10]
*RET*	receptor tyrosine kinase	2.3%	selpercatinib, pralsetinib	[5,11]
*MET*	receptor tyrosine kinase	5.5%	capmatinib, tepotinib	[5,12]
*KRAS*	GTPase	29.9%	sotorasib, adagrasib	[5,13]
*HER2*	receptor tyrosine kinase	3.8%	no targeted therapy approved	[5]

ALK–anaplastic lymphoma kinase, BRAF–proto-oncogene B-Raf, EGFR–epidermal growth factor receptor, EMA–European Medicines Agency, FDA–Food and Drug Administration, HER2–human epidermal growth factor receptor 2, KRAS–Kirsten rat sarcoma virus, LUAC–lung adenocarcinoma, MET–mesenchymal-epithelial transition factor, NSCLC–non-small-cell lung cancer, NTRK 1-3–neurotrophic tyrosine kinase type 1-3, RET–RET proto-oncogene, ROS1–ROS proto-oncogene 1

**Table 2:** Chosen studies concerning immunotherapy in NSCLC patients with driver alterations.

Study	Considered Genes	Immunotherapy Type	Endpoints	ORR
IMMUNOTARGET [22]	*EGFR*, *ALK*, *ROS1*, *KRAS*, *BRAF*, *MET*, *HER2*, *RET*	Anti-PD-1 or anti-PD-L1 mAbs	ORR, OS, PFS	*EGFR*: 12%, *ALK*: 0%, *ROS1*: 16%, *KRAS*: 26%, *BRAF*: 24%, *MET*: 16%, *HER2*: 7%, *RET*: 6%
Gainor et al. [23]	*EGFR*, *ALK*	Anti-PD-1 or anti-PD-L1 mAbs	ORR, PFS	*EGFR/ALK*: 3.6%
NCT02879994 [24]	*EGFR*	Anti-PD-1 (pembrolizumab)	ORR, PFS, OS	0%
KEYNOTE-021 [25]	*EGFR*	Anti-PD-1 (pembrolizumab) in combination with EGFR inhibitor	ORR, DLTs	41.7%
ATLANTIC [26]	*EGFR*, *ALK*	Anti-PD-L1 (durvalumab)	ORR	*EGFR/ALK*: 12.2%
Lau et al. [27]	*EGFR*, *HER2*	Anti-PD-1 or anti-PD-L1 mAbs or Anti-PD-1+Anti-CTLA-4 mAbs	ORR, PFS	*EGFR* (exon 20): 50% *EGFR* (other): 11% *HER2*: 29%
Choudhury et al. [28]	*ROS1*	Group A: Anti-PD-1 (pembrolizumab or nivolumab) or Anti-PD-L1 (atezolizumab) or an investigational agent Group B: combination of chemotherapy and ICIs	ORR, TTD	Group A: 13% Group B: 83%
Skoulidis et al. [29]	*KRAS* (plus *STK11* or *TP53*)	Anti-PD-1 or anti-PD-L1 mAbs or Anti-PD-1+Anti-CTLA-4 mAbs	ORR, PFS, OS	*KRAS* + *STK11*: 7.4% *KRAS* + *TP53*: 35.7%
Dudnik et al. [30]	*BRAF* (V600E and non-V600E)	Anti-PD-1 (pembrolizumab or nivolumab) or Anti-PD-L1 (atezolizumab)	ORR, PFS, OS	*BRAF* V600E: 25% *BRAF* non-V600E: 33%
Guisier et al. [31]	*BRAF*, *HER2*, *MET*, *RET*	Anti-PD-1 (pembrolizumab or nivolumab) or other	ORR, PFS, DoR, OS	*BRAF* V600E: 26% *BRAF* non-V600E: 35% *HER2*: 27.3% *MET*: 35.7% *RET*: 35.7%
Sabari et al. [32]	*MET*	Anti-PD-1 or anti-PD-L1 mAbs or Anti-PD-1+Anti-CTLA-4 mAbs	ORR, PFS	17%
Saalfeld et al. [33]	*HER2*	Immunotherapy in monotherapy or in combination with chemotherapy	ORR, PFS, OS	First line combination therapy: 52% Second/subsequent lines monotherapy: 16%
Hegde et al. [34]	*RET*	Anti-PD-1 or anti-PD-L1 mAbs or Anti-CTLA-4 mAbs	TTD	–
Dudnik et al. [35]	Rare targetable drivers (including *NTRK)*	Anti-PD-1 (pembrolizumab or nivolumab) or Anti-PD-L1 (atezolizumab)	ORR, PFS, OS	50% (1/2)
Zhang et al. [36]	*NTRK*	Anti-PD-1 (camrelizumab)	case study	PD
Rosen et al. [37]	*NTRK*	Anti-PD-1 or anti-PD-L1 mAbs or Anti-PD-1+Anti-CTLA-4 mAbs	ORR, PFS, OS	0%

ALK–anaplastic lymphoma kinase, BRAF–proto-oncogene B-Raf, CTLA-4–cytotoxic T lymphocyte antigen 4, DLTs–dose limiting toxicities, DoR—duration of response, EGFR–epidermal growth factor receptor, HER2–human epidermal growth factor receptor 2, KRAS–Kirsten rat sarcoma virus, mAb–monoclonal antibody, MET–mesenchymal-epithelial transition factor, NSCLC–non-small cell lung cancer, NTRK 1-3–neurotrophic tyrosine kinase type 1-3, ORR–overall response rate, OS–overall survival, PD-1–programmed death 1, PD-L1–programmed death ligand 1, PFS–progression-free survival, RET–RET proto-oncogene, ROS1–ROS proto-oncogene 1, TTD–time to treatment discontinuation.

## Data Availability

Not applicable.

## Abbreviations

ALK–anaplastic lymphoma kinase, BRAF–proto-oncogene B-Raf, CI–confidence interval, CPS–combined proportion score, CTLA-4–cytotoxic T lymphocyte antigen 4, DC–dendritic cell, DC-LAMP–dendritic cell-lysosomal associated membrane protein, DLTs–dose limiting toxicities, DoR–duration of response, EGFR–epidermal growth factor receptor, EMA–European Medicines Agency, FDA–Food and Drug Administration, HER2–human epidermal growth factor receptor 2, HR–hazard ratio, ICIs–immune checkpoint inhibitors, IHC–immunohistochemistry, irAEs–immune-related adverse events, KEAP1–kelch like ECH associated protein 1, KRAS–Kirsten rat sarcoma virus, LUAC–lung adenocarcinoma, mAb–monoclonal antibody, MET–mesenchymal-epithelial transition factor, MSI–microsatellite instability, NGS–next generation sequencing, NR–not reached, NSCLC–non-small-cell lung cancer, NTRK 1-3–neurotrophic tyrosine kinase type 1-3, ORR–overall response rate, OS–overall survival, PD–progressive disease, PD-1–programmed death 1, PD-L1–programmed death ligand 1, PFS–progression-free survival, RECIST–response evaluation criteria in solid tumors, RET–RET proto-oncogene, ROS1–ROS proto-oncogene 1, SD–stable disease, STK11–serine/threonine kinase 11, TEAEs–treatment emergent adverse events, TIL–tumor-infiltrating lymphocyte, TKIs–tyrosine kinase inhibitors, TMB–tumor mutational burden, TME–tumor microenvironment, TP53–tumor protein p53, TPS–tumor proportion score, TTD–time to treatment discontinuation, WT–wild-type

## References

[1] Sung H., Ferlay J., Siegel R.L., Laversanne M., Soerjomataram I., Jemal A., Bray F. (2021). Global Cancer Statistics 2020: GLOBOCAN Estimates of Incidence and Mortality Worldwide for 36 Cancers in 185 Countries. CA Cancer J. Clin..

[2] Lamberti G., Andrini E., Sisi M., Rizzo A., Parisi C., di Federico A., Gelsomino F., Ardizzoni A. (2020). Beyond EGFR, ALK and ROS1: Current Evidence and Future Perspectives on Newly Targetable Oncogenic Drivers in Lung Adenocarcinoma. Crit. Rev. Oncol. Hematol..

[3] Siegel R.L., Miller K.D., Fuchs H.E., Jemal A. (2021). Cancer Statistics, 2021. CA Cancer J. Clin..

[4] Shah R., Lester J.F. (2020). Tyrosine Kinase Inhibitors for the Treatment of EGFR Mutation-Positive Non–Small-Cell Lung Cancer: A Clash of the Generations. Clin. Lung Cancer.

[5] Skoulidis F., Heymach J.V. (2019). Co-Occurring Genomic Alterations in Non-Small Cell Lung Cancer and Therapy. Nat. Rev. Cancer.

[6] Russell M.C., Garelli A.M., Reeves D.J. (2022). Targeting EGFR Exon 20 Insertion Mutation in Non-Small Cell Lung Cancer: Amivantamab and Mobocertinib. Ann. Pharmacother..

[7] Peng L., Zhu L., Sun Y., Stebbing J., Selvaggi G., Zhang Y., Yu Z. (2022). Targeting ALK Rearrangements in NSCLC: Current State of the Art. Front. Oncol..

[8] Azelby C.M., Sakamoto M.R., Bowles D.W. (2021). ROS1 Targeted Therapies: Current Status. Curr. Oncol. Rep..

[9] Odogwu L., Mathieu L., Blumenthal G., Larkins E., Goldberg K.B., Griffin N., Bijwaard K., Lee E.Y., Philip R., Jiang X. (2018). FDA Approval Summary: Dabrafenib and Trametinib for the Treatment of Metastatic Non–Small Cell Lung Cancers Harboring BRAF V600E Mutations. Oncologist.

[10] Dunn B., Pharm D. (2020). Larotrectinib and Entrectinib: TRK Inhibitors for the Treatment of Pediatric and Adult Patients with NTRK Gene Fusion. J. Adv. Pract. Oncol..

[11] Cascetta P., Sforza V., Manzo A., Carillio G., Palumbo G., Esposito G., Montanino A., Costanzo R., Sandomenico C., de Cecio R. (2021). RET Inhibitors in Non-Small-Cell Lung Cancer. Cancers.

[12] Mathieu L.N., Larkins E., Akinboro O., Roy P., Amatya A.K., Fiero M.H., Mishra-Kalyani P.S., Helms W.S., Myers C.E., Skinner A.M. (2022). FDA Approval Summary: Capmatinib and Tepotinib for the Treatment of Metastatic NSCLC Harboring MET Exon 14 Skipping Mutations or Alterations. Clin. Cancer Res..

[13] Nakajima E.C., Drezner N., Li X., Mishra-Kalyani P.S., Liu Y., Zhao H., Bi Y., Liu J., Rahman A., Wearne E. (2022). FDA Approval Summary: Sotorasib for KRAS G12C-Mutated Metastatic NSCLC. Clin. Cancer Res..

[14] Ruiz-Cordero R., Devine W.P. (2020). Targeted Therapy and Checkpoint Immunotherapy in Lung Cancer. Surg. Pathol. Clin..

[15] Borghaei H., Paz-Ares L., Horn L., Spigel D.R., Steins M., Ready N.E., Chow L.Q., Vokes E.E., Felip E., Holgado E. (2015). Nivolumab versus Docetaxel in Advanced Nonsquamous Non–Small-Cell Lung Cancer. N. Engl. J. Med..

[16] Rittmeyer A., Barlesi F., Waterkamp D., Park K., Ciardiello F., von Pawel J., Gadgeel S.M., Hida T., Kowalski D.M., Dols M.C. (2017). Atezolizumab versus Docetaxel in Patients with Previously Treated Non-Small-Cell Lung Cancer (OAK): A Phase 3, Open-Label, Multicentre Randomised Controlled Trial. Lancet.

[17] Herbst R.S., Baas P., Kim D.W., Felip E., Pérez-Gracia J.L., Han J.Y., Molina J., Kim J.H., Arvis C.D., Ahn M.J. (2016). Pembrolizumab versus Docetaxel for Previously Treated, PD-L1-Positive, Advanced Non-Small-Cell Lung Cancer (KEYNOTE-010): A Randomised Controlled Trial. Lancet.

[18] Gainor J.F., Shaw A.T. (2013). Emerging Paradigms in the Development of Resistance to Tyrosine Kinase Inhibitors in Lung Cancer. J. Clin. Oncol..

[19] Akbay E.A., Koyama S., Carretero J., Altabef A., Tchaicha J.H., Christensen C.L., Mikse O.R., Cherniack A.D., Beauchamp E.M., Pugh T.J. (2013). Activation of the PD-1 Pathway Contributes to Immune Escape in EGFR-Driven Lung Tumors. Cancer Discov..

[20] Hong S., Chen N., Fang W., Zhan J., Liu Q., Kang S., He X., Liu L., Zhou T., Huang J. (2016). Upregulation of PD-L1 by EML4-ALK Fusion Protein Mediates the Immune Escape in ALK Positive NSCLC: Implication for Optional Anti-PD-1/PD-L1 Immune Therapy for ALK-TKIs Sensitive and Resistant NSCLC Patients. Oncoimmunology.

[21] Chen N., Fang W., Zhan J., Hong S., Tang Y., Kang S., Zhang Y., He X., Zhou T., Qin T. (2015). Upregulation of PD-L1 by EGFR Activation Mediates the Immune Escape in EGFR-Driven NSCLC: Implication for Optional Immune Targeted Therapy for NSCLC Patients with EGFR Mutation. J. Thorac. Oncol..

[22] Mazieres J., Drilon A., Lusque A., Mhanna L., Cortot A.B., Mezquita L., Thai A.A., Mascaux C., Couraud S., Veillon R. (2019). Immune Checkpoint Inhibitors for Patients with Advanced Lung Cancer and Oncogenic Driver Alterations: Results from the IMMUNOTARGET Registry. Ann. Oncol..

[23] Gainor J.F., Shaw A.T., Sequist L.V., Fu X., Azzoli C.G., Piotrowska Z., Huynh T.G., Zhao L., Fulton L., Schultz K.R. (2016). EGFR Mutations and ALK Rearrangements Are Associated with Low Response Rates to PD-1 Pathway Blockade in Non-Small Cell Lung Cancer: A Retrospective Analysis. Clin. Cancer. Res..

[24] Lisberg A., Cummings A., Goldman J.W., Bornazyan K., Reese N., Wang T., Coluzzi P., Ledezma B., Mendenhall M., Hunt J. (2018). A Phase II Study of Pembrolizumab in EGFR-Mutant, PD-L1+, Tyrosine Kinase Inhibitor (TKI) Naïve Patients with Advanced NSCLC. J. Thorac. Oncol..

[25] Yang J.C.H., Gadgeel S.M., Sequist L.V.D., Wu C.L., Papadimitrakopoulou V.A., Su W.C., Fiore J., Saraf S., Raftopoulos H., Patnaik A. (2019). Pembrolizumab in Combination with Erlotinib or Gefitinib as First-Line Therapy for Advanced NSCLC with Sensitizing EGFR Mutation. J. Thorac. Oncol..

[26] Garassino M.C., Cho B.C., Kim J.H., Mazières J., Vansteenkiste J., Lena H., Corral Jaime J., Gray J.E., Powderly J., Chouaid C. (2018). Durvalumab as Third-Line or Later Treatment for Advanced Non-Small-Cell Lung Cancer (ATLANTIC): An Open-Label, Single-Arm, Phase 2 Study. Lancet Oncol..

[27] Lau S.C.M., Fares A.F., Le L.W., Mackay K.M., Soberano S., Chan S.W., Smith E., Ryan M., Tsao M.S., Bradbury P.A. (2021). Subtypes of EGFR- and HER2-Mutant Metastatic NSCLC Influence Response to Immune Checkpoint Inhibitors. Clin. Lung Cancer.

[28] Choudhury N.J., Schneider J.L., Patil T., Zhu V.W., Goldman D.A., Yang S.R., Falcon C.J., Do A., Nie Y., Plodkowski A.J. (2021). Response to Immune Checkpoint Inhibition as Monotherapy or in Combination with Chemotherapy in Metastatic ROS1-Rearranged Lung Cancers. JTO Clin. Res. Rep..

[29] Skoulidis F., Goldberg M.E., Greenawalt D.M., Hellmann M.D., Awad M.M., Gainor J.F., Schrock A.B., Hartmaier R.J., Trabucco S.E., Gay L. (2018). STK11/LKB1 Mutations and PD-1 Inhibitor Resistance in KRAS-Mutant Lung Adenocarcinoma. Cancer Discov..

[30] Dudnik E., Peled N., Nechushtan H., Wollner M., Onn A., Agbarya A., Moskovitz M., Keren S., Popovits-Hadari N., Urban D. (2018). BRAF Mutant Lung Cancer: Programmed Death Ligand 1 Expression, Tumor Mutational Burden, Microsatellite Instability Status, and Response to Immune Check-Point Inhibitors. J. Thorac. Oncol..

[31] Guisier F., Dubos-Arvis C., Viñas F., Doubre H., Ricordel C., Ropert S., Janicot H., Bernardi M., Fournel P., Lamy R. (2020). Efficacy and Safety of Anti–PD-1 Immunotherapy in Patients with Advanced NSCLC with BRAF, HER2, or MET Mutations or RET Translocation: GFPC 01-2018. J. Thorac. Oncol..

[32] Sabari J.K., Leonardi G.C., Shu C.A., Umeton R., Montecalvo J., Ni A., Chen R., Dienstag J., Mrad C., Bergagnini I. (2018). PD-L1 Expression, Tumor Mutational Burden, and Response to Immunotherapy in Patients with MET Exon 14 Altered Lung Cancers. Ann. Oncol..

[33] Saalfeld F.C., Wenzel C., Christopoulos P., Merkelbach-Bruse S., Reissig T.M., Laßmann S., Thiel S., Stratmann J.A., Marienfeld R., Berger J. (2021). Efficacy of Immune Checkpoint Inhibitors Alone or in Combination with Chemotherapy in NSCLC Harboring ERBB2 Mutations. J. Thorac. Oncol..

[34] Hegde A., Andreev-Drakhlin A.Y., Roszik J., Huang L., Liu S., Hess K., Cabanillas M., Hu M.I., Busaidy N.L., Sherman S.I. (2020). Responsiveness to Immune Checkpoint Inhibitors versus Other Systemic Therapies in RET-Aberrant Malignancies. ESMO Open.

[35] Dudnik E., Bshara E., Grubstein A., Fridel L., Shochat T., Roisman L.C., Ilouze M., Rozenblum A.B., Geva S., Zer A. (2018). Rare Targetable Drivers (RTDs) in Non-Small Cell Lung Cancer (NSCLC): Outcomes with Immune Check-Point Inhibitors (ICPi). Lung Cancer.

[36] Zhang L., Liu H., Tian Y., Wang H., Yang X. (2021). A Novel NCOR2-NTRK1 Fusion Detected in a Patient of Lung Adenocarcinoma and Response to Larotrectinib: A Case Report. BMC Pulm. Med..

[37] Rosen E.Y., Goldman D.A., Hechtman J.F., Benayed R., Schram A.M., Cocco E., Shifman S., Gong Y., Kundra R., Solomon J.P. (2020). TRK Fusions Are Enriched in Cancers with Uncommon Histologies and the Absence of Canonical Driver Mutations. Clin. Cancer Res..

[38] Su P.L., Chen J.Y., Chu C.Y., Chen Y.L., Chen W.L., Lin K.Y., Ho C.L., Tsai J.S., Yang S.C., Chen C.W. (2022). The Impact of Driver Mutation on the Treatment Outcome of Early-Stage Lung Cancer Patients Receiving Neoadjuvant Immunotherapy and Chemotherapy. Sci. Rep..

[39] Sun L., Guo Y.J., Song J., Wang Y.R., Zhang S.L., Huang L.T., Zhao J.Z., Jing W., Han C.B., Ma J.T. (2021). Neoadjuvant EGFR-TKI Therapy for EGFR-Mutant NSCLC: A Systematic Review and Pooled Analysis of Five Prospective Clinical Trials. Front. Oncol..

[40] Dong Z.Y., Zhang J.T., Liu S.Y., Su J., Zhang C., Xie Z., Zhou Q., Tu H.Y., Xu C.R., Yan L.X. (2017). EGFR Mutation Correlates with Uninflamed Phenotype and Weak Immunogenicity, Causing Impaired Response to PD-1 Blockade in Non-Small Cell Lung Cancer. Oncoimmunology.

[41] Sun Y., Yang Q., Shen J., Wei T., Shen W., Zhang N., Luo P., Zhang J. (2021). The Effect of Smoking on the Immune Microenvironment and Immunogenicity and Its Relationship with the Prognosis of Immune Checkpoint Inhibitors in Non-Small Cell Lung Cancer. Front. Cell Dev. Biol..

[42] Gainor J.F., Rizvi H., Jimenez Aguilar E., Skoulidis F., Yeap B.Y., Naidoo J., Khosrowjerdi S., Mooradian M., Lydon C., Illei P. (2020). Clinical Activity of Programmed Cell Death 1 (PD-1) Blockade in Never, Light, and Heavy Smokers with Non-Small-Cell Lung Cancer and PD-L1 Expression ≥50. Ann. Oncol..

[43] Efremova M., Finotello F., Rieder D., Trajanoski Z. (2017). Neoantigens Generated by Individual Mutations and Their Role in Cancer Immunity and Immunotherapy. Front. Immunol..

[44] Krieger T., Pearson I., Bell J., Doherty J., Robbins P. (2020). Targeted Literature Review on Use of Tumor Mutational Burden Status and Programmed Cell Death Ligand 1 Expression to Predict Outcomes of Checkpoint Inhibitor Treatment. Diagn. Pathol..

[45] Schoenfeld A.J., Arbour K.C., Rizvi H., Iqbal A.N., Gadgeel S.M., Girshman J., Kris M.G., Riely G.J., Yu H.A., Hellmann M.D. (2019). Severe Immune-Related Adverse Events Are Common with Sequential PD-(L)1 Blockade and Osimertinib. Ann. Oncol..

[46] Lin J.J., Chin E., Yeap B.Y., Ferris L.A., Kamesan V., Lennes I.T., Sequist L.V., Heist R.S., Mino-Kenudson M., Gainor J.F. (2019). Brief Report: Increased Hepatotoxicity Associated with Sequential Checkpoint Inhibitor and Crizotinib Therapy in Patients with Non-Small-Cell Lung Cancer. J. Thorac. Oncol..

[47] Friedlaender A., Drilon A., Weiss G.J., Banna G.L., Addeo A. (2020). KRAS as a Druggable Target in NSCLC: Rising like a Phoenix after Decades of Development Failures. Cancer Treat. Rev..

[48] Skoulidis F., Byers L.A., Diao L., Papadimitrakopoulou V.A., Tong P., Izzo J., Behrens C., Kadara H., Parra E.R., Canales J.R. (2015). Co-Occurring Genomic Alterations Define Major Subsets of KRAS–Mutant Lung Adenocarcinoma with Distinct Biology, Immune Profiles, and Therapeutic Vulnerabilities. Cancer Discov..

[49] Skoulidis F., Li B.T., Dy G.K., Price T.J., Falchook G.S., Wolf J., Italiano A., Schuler M., Borghaei H., Barlesi F. (2021). Sotorasib for Lung Cancers with KRAS p.G12C Mutation. N. Engl. J. Med..

[50] Ghimessy A., Radeczky P., Laszlo V., Hegedus B., Renyi-Vamos F., Fillinger J., Klepetko W., Lang C., Dome B., Megyesfalvi Z. (2020). Current Therapy of KRAS-Mutant Lung Cancer. Cancer Metastasis Rev..

[51] Jänne P.A., Riely G.J., Gadgeel S.M., Heist R.S., Ou S.-H.I., Pacheco J.M., Johnson M.L., Sabari J.K., Leventakos K., Yau E. (2022). Adagrasib in Non–Small-Cell Lung Cancer Harboring a KRAS G12C Mutation. N. Engl. J. Med..

[52] Kadara H., Choi M., Zhang J., Parra E.R., Rodriguez-Canales J., Gaffney S.G., Zhao Z., Behrens C., Fujimoto J., Chow C. (2017). Whole-Exome Sequencing and Immune Profiling of Early-Stage Lung Adenocarcinoma with Fully Annotated Clinical Follow-Up. Ann. Oncol..

[53] Dong Z.Y., Zhong W.Z., Zhang X.C., Su J., Xie Z., Liu S.Y., Tu H.Y., Chen H.J., Sun Y.L., Zhou Q. (2017). Potential Predictive Value of TP53 and KRAS Mutation Status for Response to PD-1 Blockade Immunotherapy in Lung Adenocarcinoma. Clin. Cancer Res..

[54] Pore N., Wu S., Standifer N., Jure-Kunkel M., de los Reyes M., Shrestha Y., Halpin R., Rothstein R., Mulgrew K., Blackmore S. (2021). Resistance to Durvalumab and Durvalumab plus Tremelimumab Is Associated with Functional STK11 Mutations in Patients with Non–Small Cell Lung Cancer and Is Reversed by STAT3 Knockdown. Cancer Discov..

[55] Sotorasib Activity in Subjects with Advanced Solid Tumors with KRAS p.G12C Mutation (CodeBreak 101)–Full Text View–ClinicalTrials.Gov. https://clinicaltrials.gov/ct2/show/NCT04185883.

[56] Phase 2 Trial of MRTX849 Monotherapy and in Combination with Pembrolizumab for NSCLC with KRAS G12C Mutation KRYSTAL-7–Full Text View–ClinicalTrials.Gov. https://clinicaltrials.gov/ct2/show/NCT04613596.

[57] Chen D., Zhang L.Q., Huang J.F., Liu K., Chuai Z.R., Yang Z., Wang Y.X., Shi D.C., Liu Q., Huang Q. (2014). BRAF Mutations in Patients with Non-Small Cell Lung Cancer: A Systematic Review and Meta-Analysis. PLoS ONE.

[58] Spigel D.R., Schrock A.B., Fabrizio D. (2016). Total Mutation Burden (TMB) in Lung Cancer (LC) and Relationship with Response to PD-1/PD-L1 Targeted Therapies. J. Clin. Oncol..

[59] Giustini N., Bazhenova L. (2021). Recognizing Prognostic and Predictive Biomarkers in the Treatment of Non-Small Cell Lung Cancer (NSCLC) with Immune Checkpoint Inhibitors (ICIs). Lung Cancer Targets Ther..

[60] Singal G., Miller P.G., Agarwala V., Li G., Kaushik G., Backenroth D., Gossai A., Frampton G.M., Torres A.Z., Lehnert E.M. (2019). Association of Patient Characteristics and Tumor Genomics with Clinical Outcomes Among Patients with Non-Small Cell Lung Cancer Using a Clinicogenomic Database. JAMA.

[61] Amatu A., Sartore-Bianchi A., Bencardino K., Pizzutilo E.G., Tosi F., Siena S. (2019). Tropomyosin Receptor Kinase (TRK) Biology and the Role of NTRK Gene Fusions in Cancer. Ann. Oncol..

[62] Lin L., Hu X., Zhang H., Hu H. (2019). Tertiary Lymphoid Organs in Cancer Immunology: Mechanisms and the New Strategy for Immunotherapy. Front. Immunol..

[63] Rakaee M., Adib E., Ricciuti B., Sholl L.M., Shi W., Alessi J.V.M., Cortellini A., Fulgenzi C.A.M., Pinato D.J.J., Hashemi S.M. (2022). Artificial Intelligence in Digital Pathology Approach Identifies the Predictive Impact of Tertiary Lymphoid Structures with Immune-Checkpoints Therapy in NSCLC. J. Clin. Oncol..

[64] Biton J., Mansuet-Lupo A., Pécuchet N., Alifano M., Ouakrim H., Arrondeau J., Boudou-Rouquette P., Goldwasser F., Leroy K., Goc J. (2018). TP53, STK11, and EGFR Mutations Predict Tumor Immune Profile and the Response to Anti–PD-1 in Lung Adenocarcinoma. Clin. Cancer Res..

